# Acknowledgment of Reviewers

**DOI:** 10.2188/jea.JE20140203

**Published:** 2014-11-05

The following scientists assisted the journal by reviewing manuscripts during the period October 1, 2013 to September 30, 2014. We greatfully acknowledge their critical evaluation and assistance in selecting articles for publication.

**Table:** 

Adachi, Hisashi	Kurume University
Aida, Jun	Tohoku University Graduate School of Dentistry
Akasaka, Hiroshi	Sapporo Medical University School of Medicine
Akiba, Suminori	Kagoshima Univeristy
Akiyama, Yoshimitsu	Tokyo Medical and Dental University
Ando, Masahiko	Nagoya University Hospital
Ando, Yuichi	The National Institute of Public Health
Ansai, Toshihiro	Kyushu Dental University
Araki, Atsushi	Tokyo Metropolitan Geriatric Hospital
Aramaki, Eiji	Kyoto University
Arima, Hisatomi	The George Institute
Arisawa, Kokichi	Institute of Health Biosciences, the University of Tokushima Graduate School
Asao, Keiko	University of Tennessee Health Science Center
Asayama, Kei	University of Leuven
Babazono, Akira	Kyushu University
Cai, Guoxi	Research Institute for Humanity and Nature
Chaves, Paulo	Florida International University
Chihara, Dai	Aichi Cancer Center Research Institute
Doi, Takehiko	National Center for Geriatrics and Gerontology
Doi, Yasufumi	Graduate School of Medical Sciences, Kyushu University
Domijan, Ana-Marija	University of Zagreb, Faculty of Pharmacy and Biochemistry
Eguchi, Hisashi	Kitasato University School of Medicine
Ekuni, Daisuke	Okayama University
Flowers, Christopher	Emory University
Fujita, Koji	Akita University Graduate School of Medicine
Fujiyoshi, Akira	Shiga University of Medical Science
Fukuda, Yoshiharu	Yamaguchi Unversity School of Medicine
Funatogawa, Ikuko	The Institute of Statistical Mathematics
Fushimi, Kiyohide	Tokyo Medical and Dental University
Goto, Atsushi	National Center for Global Health and Medicine
Goto, Aya	Fukushima Medical University
Haaramo, Peija	National Institute for Health and Welfare
Hamajima, Nobuyuki	Nagoya University Graduate School of Medicine
Hara, Megumi	Saga University
Hashimoto, Hideki	School of Public Health, The University of Tokyo
Hashizume, Masahiro	Institute of Tropical Medicine, Nagasaki University
Hata, Jun	Graduate School of Medical Sciences, Kyushu University
Hayashi, Kunihiko	Gunma University
Hayashi, Takahito	Graduate School of Medical and Dental Sciences, Kagoshima University
Hayashino, Yasuaki	Kyoto University, Graduate School of Medicine
Higashi, Takahiro	National Cancer Center
Higashiyama, Aya	National Cerebral and Cardiovascular Center of Japan
Hirao, Susumu	The Research Institute of Tuberculosis
Hirose, Kaoru	Aichi Prefectural Institute of Public Health
Hishida, Tomoyuki	National Cancer Center Hospital East
Honda, Sumihisa	Nagasaki University Graduate School of Biomedical Sciences
Honda, Yasushi	University of Tsukuba
Honjo, Kaori	Osaka University Global Collaboration Center
Honjo, Satoshi	Fukuoka East Medical Centre
Hosono, Akihiro	Mizuho Health Center, City of Nagoya
Hosono, Satoyo	Aichi Cancer Center Research Institute
Hu, Gang	Pennington Biomedical Research Center
Huang, Jee	Hepatobiliary Division, Department of Internal Medicine
Hung, Ming-Jui	Chang Gung Memorial Hospital
Hwang, Jiyun	Sangmyung University
Ichikawa, Masao	University of Tsukuba
Ikeda, Nayu	The University of Tokyo
Imaeda, Nahomi	Nagoya Women’s University
Imamura, Fumiaki	University of Cambridge
Imano, Hironori	Graduate School of Medicine, Osaka University
Inoue, Akiomi	University of Occupational and Environmental Health, Japan
Inoue, Shigeru	Tokyo Medical University
Ishihara, Junko	Sagami Women’s University
Ishikawa, Hirofumi	Okayama University
Ito, Jun	National Center for Child Health and Development
Ito, Yuri	Osaka Medical Center for Cancer and Cardiovascular Disease
Itoh, Yasuhiko	Nippon Medical School
Jwa, Seung Chik	National Research Institute for Child Health and Development, National Center for Child Health and Development
Kabeya, Yusuke	Tokyo Saiseikai Central Hospital
Kachi, Yuko	Nippon Medical School
Kadotani, Hiroshi	Shiga University of Medical Science
Kakehashi, Masayuki	Hiroshima University
Kakizaki, Masako	Tohoku University Graduate School of Medicine
Kaku, Koki	National Defense Medical Research Institute
Kamada, Masamitsu	National Institute of Health and Nutrition
Kanatani, Kumiko	Kyoto University School of Public Health
Kanda, Hideyuki	Fukushima Medical University
Katanoda, Kota	National Cancer Center
Kato, Tuguhiko	Hiroshima University
Kawai, Masaaki	Tohoku University Graduate School of Medicine
Kawamura, Takashi	Kyoto University
Kayaba, Kazunori	Saitama Prefectural University
Kikuchi, Shogo	Aichi Medciacl University School of Medicine
Kim, Mi Kyung	College of Medicine, Hanyang University
Kim, Mi-ji	Tokyo Metropolitan Institute of Gerontology
Kobashi, Gen	National Institute of Radiological Sciences
Kobayashi, Minatsu	Otsuma Women’s University
Kobayashi, Nobumichi	Sapporo Medical University
Kohyama, Jun	Tokyo Bay Urayasu/Ichikawa Medical Center
Kojima, Masayo	Nagoya City University
Kokubo, Yoshihiro	National Cardiovascular Center
Kondo, Naoki	University of Tokyo School of Public Health
Koriyama, Chihaya	Kagoshima University
Kotani, Kazuhiko	Jichi Medical University
Kumar Dash, Paban	Defence Research and Development Establishment
Kuriki, Kiyonori	Laboratory of Public Health, School of Food and Nutritional Sciences
Kuriyama, Nagato	Kyoto Prefectural University of Medicine
Kurokawa, Naoyuki	Miyagi University of Education
Lee, Hae-Jeung	Eulji University
Li, Wei	National Center for Cardiovascular Diseases of China
Lin, Yingsong	Aichi Medical Uniersity
Liu, Jianmeng	Institute of Reproductive and Child Health, Peking University Health Science Center
Liu, Qi-Yong	National Institute for Communicable Disease Control and Prevention, Chinese Center for Disease Control and Prevention
Lv, Jun	Peking University Health Science Center
Ma, Enbo	University of Tsukuba
Mariappan, Thiruppathi	Indian Council of Medical Research
Maruyama, Shoichi	Nagoya University School of Medicine
Masamune, Atsushi	Tohoku University
Matsui, Kenji	National Cerebral and Cardiovascular Center of Japan
Matsushita, Yumi	National Center for Global Health and Medicine
Metoki, Hirohito	Tohoku University School of Medicine
Michikawa, Takehiro	National Institute for Environmental Studies
Minami, Yuko	Tohoku University School of Health Sciences
Mitani, Yoshihide	Mie University Graduate School of Medicine
Miura, Katsuyuki	Shiga University of Medical Science
Miura, Yoshihiko	Saitama Prefectural University
Miyachi, Motohiko	National Institute of Health & Nutrition
Miyake, Yoshihiro	Ehime University Graduate School of Medicine
Miyazaki, Kikuko	Kyoto University
Mizoue, Tetsuya	International Medical Center of Japan
Mori, Mitsuru	Sapporo Medical University
Morikawa, Yuko	Kanazawa Medical University
Morisaki, Naho	National Center for Child Health and Deveopment
Murakami, Yoshitaka	Shiga University of Medical Science
Muraki, Isao	Osaka Center for Cancer and Cardiovascular Diseases Prevention
Murata, Chiyoe	Hamamatsu University School of Medicine
Nagai, Masato	Fukushima Medical University
Nagata, Chisato	Gifu University Graduate School of Medicine
Nakagomi, Osamu	Nagasaki University
Nakahara, Shinji	St. Mariana University
Nakai, Satoshi	Yokohama National University
Nakai, Shigeru	Fujita Health University School of Health Sciences
Nakamura, Hiroyuki	Graduate School of Medical Science, Kanazawa University
Nakamura, Kazutoshi	Niigata University
Nakamura, Koshi	Kanazawa Medical University
Nakamura, Mieko	Hamamatsu University
Nakamura, Yasuyuki	Kyoto Women’s University
Nakamura, Yosikazu	Jichi Medical University
Nakata, Akinori	University of Occupational and Environmental Health
Nakata, Yoshio	University of Tsukuba
Nakaya, Naoki	Kamakura Women’s University
Nakayama, Takeo	Kyoto University School of Public Health
Nakazawa, Minato	Kobe University, Graduate School of Health Sciences
Ng, Chris Fook Sheng	National Institute for Environmental Studies
Niimi, Akiko	Nagoya City University Graduate School of Medical Sciences
Nishi, Nobuo	National Institute of Health and Nutrition
Nishikitani, Mariko	Kyushu University
Nishimura, Kunihiro	National Cardiovascular and Cerevral Center
Nishino, Yoshikazu	Miyagi Cancer Center Research Institute
Nishiyama, Takeshi	Nagoya City University
Noda, Hiroyuki	Harvard School of Public Health
Nojima, Masanori	Sapporo Medical University, School of Medicine
Oba, Shino	National Institute of Public Health
Ohira, Tetsuya	Osaka University
Ohsawa, Masaki	Iwate Medical University
Ojima, Toshiyuki	Hamamatsu Medical University
Okada, Masafumi	University of Tsukuba
Okamoto, Etsuji	National Institute of Public Health
Okamoto, Kazushi	Aichi Prefectural University
Okayama, Akira	Research Institute for Strategy of Prevention
Okuda, Nagako	National Institute of Health and Nutrition
Ono, Masaji	National Institute for Environmental Studies
Osaki, Yoneatsu	Tottori University
Otsuka, Rei	National Center for Geriatrics and Gerontology
Ozasa, Kotaro	RERF
Oze, Isao	Aichi Cancer Center Research Institute
Pan, An	National University of Singapore
Park, Boyoung	National Cancer Center
Park, Kyong	Yeungnam University
Pham, Truong-Minh	School of Public Health, University of Alberta
Qin, Li-Qiang	Soochow University
Sadahiro, Tomohito	Tokyo Women’s Medical University Yachiyo Medical Center
Sadakane, Atsuko	Radiation Effects Research Foundation
Saeki, Satoru	School of Medicine, University of Occupational and Environmental Health, Japan
Saijo, Yasuaki	Asahikawa Medical College
Saika, Kumiko	Center for Cancer Control and Information Services, National Cancer Center
Sairenchi, Toshimi	Dokkyo Medical University School of Medicine
Sakamoto, Wataru	Okayama University
Sakurai, Masaru	Kanazawa Medical University
Sakurai, Ryota	Tokyo Metropolitan Institute of Gerontology
Sasazuki, Shizuka	Research Center for Cancer Prevention and Screening, National Cancer Center
Sato, Miri	University of Yamanashi
Satoh, Toshihiko	Kitasato University
Sawa, Tomohiro	Tohoku University Graduate School of Medicine
Sawada, Norie	Research Center for Cancer Prevention and Screening, National Cancer Center
Sengoku, Kazuo	Asahikawa Medical College
Shima, Masayuki	Hyogo College of Medicine
Shimazu, Taichi	National Cancer Center
Shimozuma, Kojiro	Ritsumeikan University
Shin, Aesun	Seoul National University College of Medicine
Shinkai, Shoji	Tokyo Metropolitan Institute of Gerontology
Shobugawa, Yugo	Niigata University
Shuko, Nagai	Kyoto University
Sone, Hirohito	University of Tsukuba Institute of Clinical Medicine
Song, Minkyo	Seoul National University College of Medicine
Sonobe, Tomoyoshi	Japanese Red Cross Medical Center
Sugimura, Haruhiko	Hamamatsu Medical University
Sugiyama, Daisuke	Keio University
Sugiyama, Hiromi	Radiation Effects Research Foundation
Sumi, Ayako	Sapporo Medical University School of Medicine
Suzuki, Kohta	University of Yamanashi, Interdisciplinary Graduate School of Medicine and Engineering
Tabara, Yasuharu	Kyoto University Graduate School of Medicine
Tabuchi, Takahiro	Osaka Medical Center for Cancer and Cardiovascular Disease
Taira, Naruto	Okayama University
Tajima, Kazuo	Mie University
Takachi, Ribeka	Niigata University Graduate School of Medical and Dental Sciences
Takagi, Daisuke	The University of Tokyo
Takahashi, Kunihiko	Nagoya University Graduate School of Medicine
Takahashi, Masaya	Japan NIOSH
Takano, Takehito	Tokyo Medical and Dental University
Takashima, Naoyuki	Shiga University of Medical Science
Takatorige, Toshio	Kansai University
Takeuchi, Ayano	National Institute for Environmental Studies
Tamakoshi, Akiko	Hokkaido University Graduate School of Medicine
Tamashiro, Hiko	Hokkaido University
Tanabe, Naohito	Niigata University Graduate School of Medical and Dental Sciences
Tanaka, Junko	Hiroshima University Graduate School of Biomedical Sciences
Tanaka, Keiko	Faculty of Medicine, Fukuoka University
Tanaka, Keitaro	Saga University
Tanaka, Noriko	NCGM
Tanaka, Shiro	Kyoto University
Tanihara, Shinichi	Fukuoka University
Tanimura, Susumu	Hyogo College of Medicine
Teramukai, Satoshi	Kyoto Prefectural University of Medicine
Tierney, ES Selamet	Stanford Medicine School of Medicine
Tokudome, Yuko	Nagoya University of Arts and Sciences
Tomata, Yasutake	Tohoku University Graduate School of Medicine
Tsuboya, Toru	Tohoku University Graduate School of Medicine
Tsukuma, Hideaki	Osaka Medical Center for Cancer and Cardiovascular Disease
Tsutsumi, Akizumi	UOEH
Turin, Tanvir	University of Calgary
Uchigata, Yasuko	Tokyo Women’s Medical University School of Medicine
Ueda, Kayo	National Institute for Environmental Studies
Ueda-Ballmer, Michiko	Syracuse University
Uemura, Kazuki	National Center for Geriatrics and Gerontology
Umesawa, Mitsumasa	Center for Medical Sciences
Wada, Keiko	Gifu University Graduate School of Medicine
Wagatsuma, Yukiko	University of Tsukuba
Wang, Peiyu	Peking University
Wang, Qingsheng	Tianjin Cancer Institute and Hospital, Tianjin Medical University
Washio, Masakazu	St. Mary’s College
Watanabe, Makoto	National Cardiovascular Center
Watanabe, Ryo	Hitotsubashi University
Watanabe, Shuichiro	J. F. Oberlin University
Won, Young-joo	National Cancer Center
Xiang, Yong-Bing	Shanghai Cancer Institute
Yamaguchi, Takuhiro	Tohoku University
Yamaji, Taiki	National Cancer Center
Yamamoto, Seiichiro	National Cancer Center
Yamazaki, Shin	Kyoto University School of Public Health
Yasuda, Yoshinari	Nagoya University
Yatsuya, Hiroshi	Fujita Health University
Yokomichi, Hiroshi	University of Yamanashi
Yokoyama, Yukari	Nihon Fukushi University
Yonemoto, Naohiro	National Center of Neurology and Psychiatry
Zhao, Fang-Hui	National Cancer Center & Cancer Hospital, Chinese Academy of Medical Sciences & Peking Union Medical College
Zhou, Maigeng	University of Washington

